# Spatial data of *Ixodes ricinus* instar abundance and nymph pathogen prevalence, Scandinavia, 2016–2017

**DOI:** 10.1038/s41597-020-00579-y

**Published:** 2020-07-16

**Authors:** Lene Jung Kjær, Kirstine Klitgaard, Arnulf Soleng, Kristin Skarsfjord Edgar, Heidi Elisabeth H. Lindstedt, Katrine M. Paulsen, Åshild Kristine Andreassen, Lars Korslund, Vivian Kjelland, Audun Slettan, Snorre Stuen, Petter Kjellander, Madeleine Christensson, Malin Teräväinen, Andreas Baum, Laura Mark Jensen, René Bødker

**Affiliations:** 1 Department of Veterinary and Animal Sciences, Faculty of Health and Medical Sciences, University of Copenhagen, Frederiksberg, Denmark; 2 Department for Diagnostics and Scientific Advice, National Veterinary Institute, Technical University of Denmark, Lyngby, Denmark; 3 Department of Pest Control, Norwegian Institute of Public Health, Oslo, Norway; 4 Department of Virology, Norwegian Institute of Public Health, Oslo, Norway; 5 Department of Production Animal Clinical Sciences, Norwegian University of Life Sciences, Oslo, Norway; 6 Department of Natural Sciences, University of Agder, Kristiansand, Norway; 7 Sørlandet Hospital Health Enterprise, Research Unit, Kristiansand, Norway; 8 Department of Production Animal Clinical Sciences, Section of Small Ruminant Research, Norwegian University of Life Sciences, Sandnes, Norway; 9 Department of Ecology, Grimsö Wildlife Research Station, Swedish University of Agricultural Sciences, Riddarhyttan, Sweden; 10 Department of Applied Mathematics and Computer Science, Technical University of Denmark, Lyngby, Denmark

**Keywords:** Entomology, Bacterial infection, Ecological epidemiology

## Abstract

Ticks carry pathogens that can cause disease in both animals and humans, and there is a need to monitor the distribution and abundance of ticks and the pathogens they carry to pinpoint potential high risk areas for tick-borne disease transmission. In a joint Scandinavian study, we measured *Ixodes ricinus* instar abundance at 159 sites in southern Scandinavia in August-September, 2016, and collected 29,440 tick nymphs at 50 of these sites. We additionally measured abundance at 30 sites in August-September, 2017. We tested the 29,440 tick nymphs in pools of 10 in a Fluidigm real-time PCR chip to screen for 17 different tick-associated pathogens, 2 pathogen groups and 3 tick species. We present data on the geolocation, habitat type and instar abundance of the surveyed sites, as well as presence/absence of each pathogen in all analysed pools from the 50 collection sites and individual prevalence for each site. These data can be used alone or in combination with other data for predictive modelling and mapping of high-risk areas.

## Abstract (summary)

**Table:** 

Measurement(s)	pathogen • Ixodida • Abundance • tick instar abundance
Technology Type(s)	real-time PCR • Manual Count
Factor Type(s)	geographic location • time of data collection
Sample Characteristic - Organism	Ixodes ricinus
Sample Characteristic - Environment	forested area • meadow ecosystem
	Kingdom of Denmark • Kingdom of Norway • Sweden

Machine-accessible metadata file describing the reported data: 10.6084/m9.figshare.12523133

## Background & Summary

Tick-borne pathogens have become more prevalent over the last decades^1–7^ causing disease in humans and animals world-wide. Incidences of tick-borne diseases such as Lyme borreliosis have increased in many regions – including Scandinavia^8,9^. Left untreated, Lyme borreliosis can pose serious health threats to humans. Other tick-borne diseases, affecting humans and animals such as anaplasmosis (caused by *Anaplasma phagocytophilumn)*, relapsing fever (caused by *Borrelia miyamotoi)*, babesiosis (caused by *Babesia* spp.), rickettsiosis (caused by *Rickettsia* spp.) and infections by *Neoehrlichia mikurensis*, are also on the rise^10–13^. As climate has changed over the past decades, there is great concern that a warmer climate has an impact on increased incidence of vector-borne diseases as vectors might expand their distribution to more northern climates in Scandinavia^8,14^. Human behaviour evidently affects the risk of exposure to ticks and their pathogens, but multiple studies have shown how tick abundance affect not only human tick-exposure but also the prevalence of pathogens harboured in ticks^15–21^. More effective control and prevention of these diseases requires a better understanding of factors affecting vectors and their associated pathogens. Hence, there is a great need for data-based analyses and models that can identify the main drivers of vector abundance and distribution as well as drivers of disease transmission. Developing predictive models for larger areas requires underlying data, which can oftentimes be economically and logistically unfeasible. Free access to data from multiple studies would not only support any large-scale modelling attempt but also act as a reference to future repeated surveys, and thus sharing of data would be of great value to the scientific community.

In Europe there are numerous studies on ticks and their pathogens that have been reported in scientific journals^3–5,9,11,20,22–30^, and some of these studies have also made data accessible through a free repository^20,31–33^. However, this is only a fraction of the published data, and finding data on tick abundance and tick-borne pathogen prevalence from a large number of sites for use in risk assessments and modelling, requires a thorough review of the immense amount of papers reporting these data, as for example was done by Estrada-Peña *et al*. both for European and South American tick data^26,33–35^. Common and free access to data on tick abundance and tick-borne pathogen prevalence would greatly benefit epidemiological research and sharing of data should always be encouraged. Therefore, we have created a public database for tick abundance and tick-borne pathogen prevalence in the figshare repository and describe the collected data in this manuscript.

The data is part of a multi-national study on ticks in southern Scandinavia. The tick abundance and prevalence data described here, was obtained using a standardised stratified design, comparable between the participating countries. The data consist of tick instar abundance measured from a total of 159 sites in 2016 and 30 sites in 2017 in Denmark, Norway and Sweden and constitute 75.6 km’s of flagged transects. We furthermore collected tick nymphs at 50 of the 159 surveyed sites, counting a total of 24,667 ticks. We used a Fluidigm real-time PCR chip to screen for 17 different pathogens and 2 pathogen groups expected to be in the region (Table 1) and furthermore screened for 3 different tick-species that have been observed in various parts of Scandinavia; *Ixodes ricinus*, *I. persulcatus*, and *Dermacentor reticulatus*. The dataset of 50 georeferenced sites of approximately 60 pools of ten nymphs each tested for 17 pathogens, 2 pathogen groups and 3 tick species thus consist of approximately 66,000 individual PCR tests. The field study was conducted within a short time-frame, from August to September in both 2016 and 2017, thus any differences between measures of abundance and pathogen prevalence within the same year is likely due to geographical differences within the region.

**Table 1:** Pathogens and tick species included in the real-time PCR assay.

Species
*Borrelia miyamotoi*, *B. burgdorferi* sensu lato *B. afzelii*, *B. burgdorferi* sensu strictu, *B. garinii*, *B. lusitaniae*, *B. spielmanii*, *B. valaisiana*
*Anaplasma phagocytophilum*
*Neoehrlichia mikurensis*
The spotted fever group (SFG) *Rickettsiae* *Rickettsia helvetica*
*Francisella tularensis*
*Coxiella burnetii*
*Babesia canis*, *Babesia divergens*, *Babesia microti*, *Babesia venatorum*
*Bartonella henselae*
*Ixodes ricinus*, *Ixodes persulcatus*, *Dermacentor reticulatus*

The abundance data has previously been used to model the geographical distribution of *I. ricinus* nymphs^36^ and abundance of *I. ricinus* larva and nymphs^37^ in Scandinavia. This data along with the aggregated data on pathogen prevalence can be helpful in assessing tick distribution and tick-borne pathogen prevalence in southern Scandinavia and thereby assess human and animal disease risk. The data alone, or in combination with other data, can also be used in R_0_ models, disease mapping, and predictive modelling.

## Methods

### Study region

**Fig. 1:**
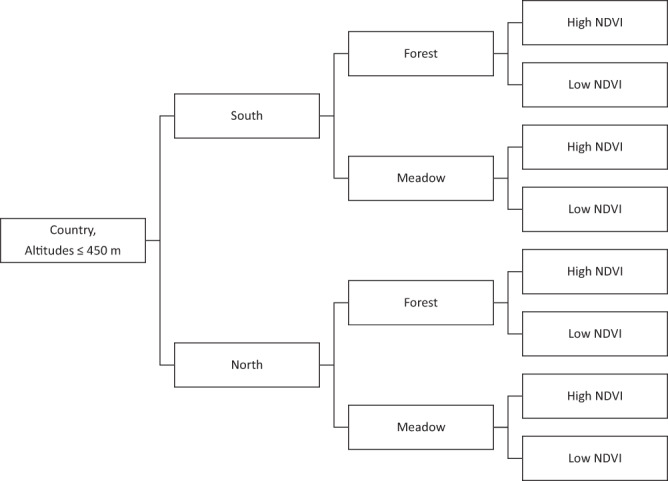
Stratification scheme Each country was divided into equally sized north and south strata. NDVI depicts the maximum normalized difference vegetation index. Forest and meadow classes are based on Corine Land Cover class definitions^38^. Forest includes the cover types: broad-leaved forest, coniferous forest and mixed forest. Meadow includes: land principally occupied by agriculture with significant areas of natural vegetation, natural grasslands, moors and heathland, and transitional woodland-shrub. All other land cover classes and altitudes >450 m (due to expected low tick abundance or complete absence) were excluded from the stratification and were not sampled for ticks.

The stratification of the study region has been described in Kjær *et al*.^36^ and Kjær *et al*.^37^, but a short description follows. The study region encompassed all of Denmark, southern Norway and south-eastern Sweden. After dividing each national study region into a northern and a southern part of equal sizes, we used 1 km^2^ resolution land cover data from Corine^38^, to divide each 1 × 1 km pixel into forest, meadow and “other” habitats. Only forests and meadow habitats were selected as potential tick habitats. For each forest and meadow pixel, we used Fourier processed satellite imagery of the maximum normalized difference vegetation index^39^ (NDVI, 1 km^2^ resolution), to further divide these habitat pixels into high NDVI (NDVI values above the median value) and low NDVI (equal to or below the median NDVI value). An overview of the stratification scheme can be found in Fig. 1.

### Site selection

We randomly selected sites in Denmark, Norway and Sweden using R 3.4.2^40^ (sampleStratified in the raster^41^ package). Our original aim was to both measure instar abundance and collect 600 tick nymphs from 30 1^st^ priority sites consisting of 80% forest and 20% meadow in each country^36^. To account for potential difficulties collecting the required number of nymphs at some of the sites, we created 10 alternative sites with the same stratification score for each of the 30 first priority sites in each country, where these 10 alternative sites were ranked after smallest distance to the original site. To account for difficulties collecting enough tick nymphs in meadow sites, we also created 10 alternative forest sites for each meadow site with the same NDVI score. We also created 20 random sites (with 10 alternatives as above) along the Oslo Fjord in Norway, as we were interested in investigating ticks in that region.

For logistical reasons, we needed to ascertain whether it was possible to collect 600 tick nymphs within a reasonable time frame at a given site. Thus, we defined a measure of effort as the amount of nymphs collected by one person during 30 minutes. We determined that a site should allow collection of 600 nymphs in 10 man hours, and thus one person should be able to collect 30 nymphs in 30 minutes. If the number of nymphs collected was below 30, no attempt was made to collect 600 tick nymphs for pathogen testing and an alternative site was explored to obtain the 600 nymphs. Tick instar abundance was, however, measured at all sites, which could result in abundance measures from more than 30 sites per country.

### Field study

**Fig. 2:**
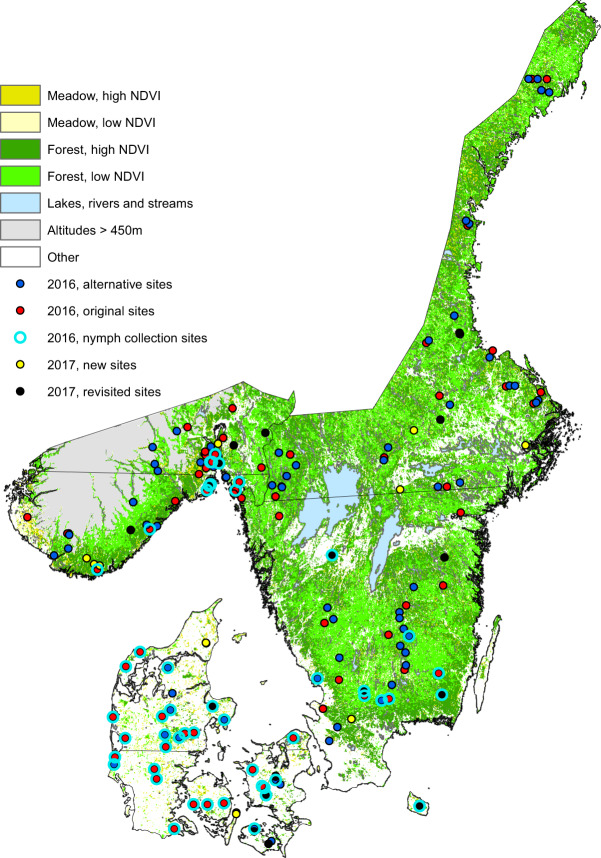
Study region. The study region with the 159 sites from 2016 divided into original sites (red), alternative sites (blue), nymph collection sites (open cyan circles) as well as the revisited sites in 2017 (black) and the new sites (yellow) from 2017. Each country study region is divided into equally sized north and south strata, depicted by the black line. Habitat definitions are found on Fig. 1. NDVI = Normalized difference vegetation index. Similar maps without nymph collection sites depicted have been published elsewhere^36,37^.

To measure tick abundance, we used two 100 m transects, one facing north and one facing east. We then “dragged”^42^ a white flannel cloth (1.05 × 1.15 m with lead weights in one end) along each transect in both directions (a total of 400 m’s) and counted and removed tick instar every 50 meters. We measured all tick instar abundance between 15 August and 30 September, 2016 within the hours of 11–16. After measuring abundance at a site, we used our preciously described effort measure to gauge whether it was feasible to collect 600 tick nymphs at the site. If the site was deemed feasible, we collected the tick nymphs, placed them in tubes and stored them on dry ice. When returning from the field, we stored the ticks at −80 °C until use. Within the allocated collection time period, we obtained abundance measures for 37 sites in Denmark, 47 sites in Norway and 75 sites in Sweden. Out of these sites, we collected approximately 600 tick nymphs from 30 sites in Denmark, 11 sites in Norway and 9 sites in Sweden (Fig. 2). Due to counting- and handling errors, we had less than 600 tick nymphs from 6 sites in Denmark and from 5 sites in Sweden, resulting in 29,440 collected tick nymphs in total. In 2017, we additionally measured abundance at 10 sites in each country, using the exact same procedure as in 2016 (August-September, between 11–16 hours). Six of these sites were previously sampled in 2016, and we chose the three sites with the lowest and the highest 2016 nymph abundance per country (excluding zeroes). We selected nymph abundance to choose the six sites in each country as nymphs were more evenly distributed than other instars. The remaining four 2017 sites were subjectively chosen by researchers in each country (Fig. 2).

### Laboratory methods

#### DNA extraction

We aggregated the tick nymphs into pools of 10, and washed them for 5 min in 70% ethanol and then for 2 × 5 min in sterile water. We homogenized the ticks with three 3-mm Tungsten beads (Qiagen) using a TissueLyser II (Qiagen, Hilden, Germany) for 2 × 2.5 min at 25 Hz in a mixture of 75 µl Incubation buffer (D920, Promega, Madison, Wisconsin, USA) and 75 µl Lysis buffer (MC501, Promega, Madison, Wisconsin, USA). We briefly centrifuged the samples at 10,000 × g for 60 s and added 30 µl Proteinase K. After an overnight incubation of the samples at 56 °C, we added 300 µl Lysis buffer (MC501, Promega, Madison, Wisconsin, USA) and vortexed the samples for a short time. We then extracted Genomic DNA, using the Maxwell 16 LEV Blood DNA kit (Promega, Madison, Wisconsin, USA) on a Maxwell^®^16 Instrument.

#### Screening of tick-borne pathogens by real-time PCR

We performed high-throughput microfluidic real-time PCR, using the BioMark real-time PCR system (Fluidigm, San Francisco, California, USA). We applied the 192.24 dynamic arrays with 22 pathogen primers, as well as one positive *E. coli* control^5^ and one negative water control. We used primers for bacterial and parasitic tick-borne pathogens previously identified in the most common tick species, *I. ricinus*, from Scandinavia, and also included common tick-borne pathogens found throughout Europe^5^. To screen for different tick species, we used primers for *I. ricinus*, *I. persulcatus*, and *D. reticulatus;* tick species observed in various regions of Scandinavia^43,44^. All pathogens targeted in the PCR assays are listed in Table 1. We pre-amplified the DNA with 2.5 μL TaqMan PreAmp Master Mix (2X), 1.2 μL pooled primer mix (0.2X) and 1.3 μL of tick DNA (total volume of 5 μL). The PCR conditions for pre-amplification were as follows: one cycle at 95 °C for 10 min, 14 cycles at 95 °C for 15 s and 4 min at 60 °C. We watered down the pre-amplified DNA 5 times and then performed real-time PCRs, using FAM- and black hole quencher (BHQ1)-labeled TaqMan probes with TaqMan Gene Expression Master Mix (according to manufacturer instructions, Applied Biosystems, Foster City, California, USA). We ran thermal cycling in the following sequence: 50 °C for 2 min, 95 °C for 10 min, 40 cycles at 95 °C for 15 s, and 60 °C for 15 s. We used the BioMark real-time PCR system for data acquisition and used the Fluidigm real-time PCR Analysis software to analyse the data and obtain crossing point (CP) values.

We considered a PCR run to be valid (Pass/Fail) when all water controls were negative, all *E. coli* controls were positive, amplification curves were accepted by Fluidigm’s algorithm for ideal curves and CP values were ≤28. Sensitivity and specificity of the test have been discussed previously^5,11,45^.

#### Pathogen prevalence

We estimated the individual-level pathogen prevalence at each site based on the number of positive pools and number of examined pools of ten nymphs each using method 3 from Cowling *et al*.^46^, assuming 100% test sensitivity and specificity. Exact confidence limits (CIs) were calculated based on binomial theory^46^.

All of our pools were positive for *I. ricinus* and negative for *I. persulcatus* and *D. reticulatus*^47^. We did not detect *F. tularensis*, *C. burnetii*, *B. canis* or *B. henselae* in any of the pools, and these data are omitted from the published database.

## Data Records

The database consists of 3 csv files^48^. The 3 files can be linked with each other through the SiteID variable, which is a unique identifier for each site.

A. The tick instar abundance table has 12 columns: (1) the unique site ID, (2) the country where the site is situated, (3) the date of measurement, (4) the site selection criteria, (5) the longitude of the site, (6) the latitude of the site, (7) the habitat type of the site, (8) the total number of larva counted from both transects, (9) the total number of nymphs counted from both transects, (10) the total number of adult females counted from both transects, (11) the total number of adult males counted from both transects, (12) the total number of tick nymphs collected and used for pathogen screening.
B. The pathogen pool results table has 19 columns: (1) the country where the site is situated, (2) the unique site ID, (3) The pool number for the particular site, (4) the Pass/Fail test result for *B. miyamotoi*, (*5*) the Pass/Fail test result for *B. burgdorferi* sensu lato, (6) the Pass/Fail test result for *B. afzelii*, (7) the Pass/Fail test result for *B. burgdorferi* sensu strictu, (8) the Pass/Fail test result for *B. garinii*, (9) the Pass/Fail test result for *B. lusitaniae*, (10) the Pass/Fail test result for *B. spielmanii*, (11) the Pass/Fail test result for *B. valaisiana*, (12) the Pass/Fail test result for *A. phagocytophilum*, (13) the Pass/Fail test result for *B. divergens*, (14) the Pass/Fail test result for *B. microti*, (15) the Pass/Fail test result for *B. venatorum*, (16) the Pass/Fail test result for *N*. *mikurensis*, (17) the Pass/Fail test result for *R. helvetica*, (18) the Pass/Fail test result for SFG *Rickettsiae*, (19) the tick species found within the pool.
C. The pathogen individual nymph prevalence table has 17 columns: (1) the country where the site is situated, (2) the unique site ID, (3) the individual prevalence in percent along with exact confidence limits for *B. miyamotoi*, (*4*) the individual prevalence in percent along with exact confidence limits for *B. burgdorferi* sensu lato, (5) the individual prevalence in percent along with exact confidence limits for *B. afzelii*, (6) the individual prevalence in percent along with exact confidence limits for *B. burgdorferi* sensu strictu, (7) the individual prevalence in percent along with exact confidence limits for *B. garinii*, (8) the individual prevalence in percent along with exact confidence limits for *B. lusitaniae*, (9) the individual prevalence in percent along with exact confidence limits for *B. spielmanii*, (10) the individual prevalence in percent along with exact confidence limits for *B. valaisiana*, (11) the individual prevalence in percent along with exact confidence limits or *A. phagocytophilum*, (12) the individual prevalence in percent along with exact confidence limits for *B. divergens*, (13) the individual prevalence in percent along with exact confidence limits for *B. microti*, (14) the individual prevalence in percent along with exact confidence limits for *B. venatorum*, (15) the individual prevalence in percent along with exact confidence limits for *N*. *mikurensis*, (16) the individual prevalence in percent along with exact confidence limits for *R. helvetica*, (17) the individual prevalence in percent along with exact confidence limits for SFG *Rickettsiae*.

## Technical Validation

### Field study

We held several meetings to discuss the standardisation of measuring tick abundance, and a video demonstrating the “dragging” method was shared with each researcher in the group to ensure that the collections were standardised as much as possible. We furthermore ensured that each country used the same fabric and same fabric size of the cloth used for “dragging”, by having these cloths produced and made by one partner for all 3 countries.

### Laboratory methods

The Fluidigm real-time PCR method has been validated through numerous studies, particularly studies pertaining to tick pathogens^5,11,22,23,43,45^.
