# Author Correction: Spatial data of *Ixodes ricinus* instar abundance and nymph pathogen prevalence, Scandinavia, 2016–2017

**DOI:** 10.1038/s41597-020-00606-y

**Published:** 2020-08-07

**Authors:** Lene Jung Kjær, Kirstine Klitgaard, Arnulf Soleng, Kristin Skarsfjord Edgar, Heidi Elisabeth H. Lindstedt, Katrine M. Paulsen, Åshild Kristine Andreassen, Lars Korslund, Vivian Kjelland, Audun Slettan, Snorre Stuen, Petter Kjellander, Madeleine Christensson, Malin Teräväinen, Andreas Baum, Laura Mark Jensen, René Bødker

**Affiliations:** 1 Department of Veterinary and Animal Sciences, Faculty of Health and Medical Sciences, University of Copenhagen, Frederiksberg, Denmark; 2 Department for Diagnostics and Scientific Advice, National Veterinary Institute, Technical University of Denmark, Lyngby, Denmark; 3 Department of Pest Control, Norwegian Institute of Public Health, Oslo, Norway; 4 Department of Virology, Norwegian Institute of Public Health, Oslo, Norway; 5 Department of Production Animal Clinical Sciences, Norwegian University of Life Sciences, Oslo, Norway; 6 Department of Natural Sciences, University of Agder, Kristiansand, Norway; 7 Sørlandet Hospital Health Enterprise, Research Unit, Kristiansand, Norway; 8 Department of Production Animal Clinical Sciences, Section of Small Ruminant Research, Norwegian University of Life Sciences, Sandnes, Norway; 9 Department of Ecology, Grimsö Wildlife Research Station, Swedish University of Agricultural Sciences, Riddarhyttan, Sweden; 10 Department of Applied Mathematics and Computer Science, Technical University of Denmark, Lyngby, Denmark

Correction to: *Scientific Data*10.1038/s41597-020-00579-y, published online 16 July 2020

Following publication of this Data Descriptor, it was found that the number of ticks collected was incorrectly stated in the Background & Summary section of the text. The correct number of ticks collected was 29,440. This error has been corrected in both the HTML and PDF versions of this Data Descriptor.

In the original figshare dataset, the sites O-035, O-074 and O-078 erroneously had number of ticks collected set to zero, this has been amended. Additionally, ticks were not collected at revisited sites as originally indicated, and this has also been amended in the related figshare record.

